# The Role of Regulatory T Cells and TH17 Cells in Multiple Myeloma

**DOI:** 10.1155/2012/293479

**Published:** 2012-03-27

**Authors:** Walter M. T. Braga, Djordje Atanackovic, Gisele W. B. Colleoni

**Affiliations:** ^1^Disciplina de Hematologia e Hemoterapia, Departamento de Oncologia Clínica e Experimental, Campus São Paulo, UNIFESP, Rua Botucatu 740, 3o Andar, 04023-900 São Paulo, SP, Brazil; ^2^Department of Oncology and Hematology and Department of Stem Cell Transplantation, University Medical Center Hamburg-Eppendorf, 20246 Hamburg, Germany

## Abstract

The development of multiple myeloma (MM) involves a series of genetic alterations and changes in the bone marrow microenvironment, favoring the growth of the tumor and failure of local immune control. Quantitative and functional alterations in CD4^+^ and CD8^+^ T cells have been described in MM. The balance between T regulatory cells (Treg) and T helper (Th) 17 cells represents one essential prerequisite for maintaining anti-tumor immunity in MM. Tregs play an important role in the preservation of self-tolerance and modulation of overall immune responses against infections and tumor cells. In MM patients, Tregs seem to contribute to myeloma-related immune dysfunction and targeting them could, therefore, help to restore and enhance vital immune responses. Th17 cells protect against fungal and parasitic infections and participate in inflammatory reactions and autoimmunity. The interplay of TGF-**β** and IL-6, expressed at high levels in the bone marrow of myeloma patients, may affect generation of Th17 cells both directly or via other pro-inflammatory cytokines and thereby modulate antitumor immune responses. A detailed analysis of the balance between Tregs and Th17 cells seems necessary in order to design more effective and less toxic modes of immunotherapy myeloma which still is an uncurable malignancy.

## 1. Introduction

 Multiple myeloma (MM) is a clonal B-cell malignancy characterized by an accumulation of mature plasma cells in the bone marrow, leading to bone destruction and failure of normal hematopoiesis [1]. MM remains an incurable disease even with the use of proteasome inhibitor bortezomib, immunomodulatory drugs (thalidomide or lenalidomide), and high-dose chemotherapy with autologous stem cell transplantation (SCT), as part of first line therapy [2]. The result of new US Food-and-Drug-Administration- (FDA-) approved treatments in the past 7 years was a doubling of patient survival from 3-4 to 7-8 years [2]. The paradigm of drug development in MM has been targeting tumor cells in their BM microenvironment [2].

The development of MM involves a series of genetic alterations and changes in the BM microenvironment, favoring the growth of the tumor and the collapse of local immune control. Classically, MM is characterized by different stages of disease which, although not discernible in every patient, progress from monoclonal gammopathy of uncertain significance (MGUS) though to active disease, a plateau phase, relapsing disease, and finally, resistant disease [3].

Tumor cells and stromal cells interact via adhesion molecules and cytokine networks to simultaneously promote tumour cell survival, drug resistance, angiogenesis, and disordered bone metabolism. A number of immunologically active compounds are increased including transforming growth factor-beta (TGF-*β*), IL-10, IL-6, and vascular endothelial growth factor (VEGF). Cellular immune defects in MM characterized by decrease in CD19 B cells, CD4, and CD8 cells have been shown to negative correlate with survival, indicating a potential positive relationship between cellular components of immune system and disease control [3]. 

Accordingly, a significant impairment of T-cell function has been described for patients with MM and patients with MGUS. Although quantitative and functional alterations in CD4 and CD8 cells have been demonstrated in MM and MGUS, the biologic basis for these abnormalities remains unclear [4].

CD4^+^CD25^+^FOXP3^+^ T regulatory (Treg) cells play an important role in the maintenance of self-tolerance and the modulation of overall immune responses against infections and tumor cells. The abnormal Treg activity in MM patients could, on the other hand, contribute to the myeloma-related immune dysfunction targeting them that could, therefore, help to restore and enhance vital immune responses [1].

T helper 17 (Th17) cells, a recently described CD4^+^ T-cell subset, protect hosts against fungal and parasitic infections and participate in inflammatory reactions and autoimmunity [5]. The role of Th17 cells in tumor pathogenesis is still not well defined. However, it seems possible that the balance between Treg and Th17 cells is particularly essential for maintaining homeostasis of antitumor immunity [5]. 

## 2. T Regulatory Cells

 Natural Tregs develop during normal T-cell maturation in the thymus and are responsible for tolerance against self-antigens. They represent 5% to 10% of the CD4^+^ cells compartment in the peripheral blood [1]. Tregs express CD4 and CD25 surface antigens as well as CTLA-4, GITR, CD103, CD62L, CD69, CD134, CD71, CD54, and CD45RA. The suppressive activity of Treg cells is associated with the overexpression of *FOXP3*, a member of the forkhead/winged helix family, which acts as a transcriptional repressor [1].

Over the last few years, extensive literature has been published on Tregs in the context of malignancy, infections, and autoimmunity. Some studies have shown that the number of CD4^+^CD25^+^ cells is increased in the peripheral blood and bone marrow of MGUS and MM patients compared with controls, suggesting that Tregs might play a role in undermining anti-infectious and antimyeloma immunity in this hematological malignancy [3, 4].

Cytotoxic T lymphocyte-associated antigen 4 (CTLA-4) is a coinhibitory molecule expressed by activated T cells and a subset of regulatory T cells. CTLA-4 is of primary importance in maintaining immune homeostasis by downregulating T-cell signaling costimulatory pathways and contributing to tolerance to self-antigens [6].

Two monoclonal antibodies against human CTLA-4, ipilimumab and tremelimumab, have been reported to elicit objective and durable responses against tumor cells in clinical trials. However, the functional impact of anti-CTLA-4 therapy on human immune responses to tumor antigens is not yet fully understood [6].

Recent studies have conducted extensive immunologic monitoring on a panel of patients selected from a large cohort of metastatic melanoma patients treated with ipilimumab. Late onset of complete or partial remission was noted, occurring after more than 12 weeks of treatment in the majority of responding patients. Some patients demonstrated overt progression before eventually responding or showing disease stabilization during ipilimumab treatment. This phenomenon of clinical progression followed by regression represents a response pattern atypical for cytotoxic therapies. Unfortunately, while clinical trials have shown that anti-CTLA-4 antibody therapy can have potent antitumor effects in a subset of metastatic melanoma patients, there have been few studies of its functional impact on human antigen-specific immune responses in other tumors, such as MM [6].

## 3. Th17 Cells

 Th17 cells differentiate in the presence of interleukin-6 (IL-6), IL-1, IL-21, and IL-23, with or without transforming growth factor-beta (TGF-*β*), and produce IL-17 and IL-22. Activated Th17 cells produce most of the IL-17, but CD8^+^ T cells, natural killer cells, and neutrophils also produce variable amounts of IL-17. IL-17 induces expression of a number of chemokines and cytokines including IL-6, TGF-*β*, granulocyte-colony stimulating factor or granulocyte-macrophage-colony stimulating factor, matrix metalloproteinase, and intercellular adhesion molecule-1 in a variety of cell types, including bone marrow stromal cells [5].

One of the Th17-specific transcription factors is the orphan nuclear receptor ROR*γ*. Its specific isoform ROR*γ*t is selectively expressed by Th17 cells and is regulated by STAT3. Overexpression of *ROR*γ*t *promotes Th17 differentiation when Th1 and Th2 development is inhibited. However, a defective ROR*γ*t does not result in the complete abolishment of Th17 differentiation or the total inhibition of autoimmunity, suggesting that additional factors are involved [7].

A significant body of information has emerged supporting a critical role of immune cells (and associated cytokines) as well as immune dysregulation in MM. The interplay of TGF-*β* and IL-6, which are both expressed at high levels in MM bone marrow, may affect generation of Th17 cells both directly or via other proinflammatory cytokines and thereby modulate antitumor immune responses [5].

## 4. The Reciprocal Relationship between Th17 Cells and Tregs

 Treg and Th17 developmental programs are reciprocally interconnected: upon TCR stimulation and a naive T cell can be driven to express Foxp3 and become a Treg cell in the presence of TGF-*β*. However, in the presence of TGF-*β* plus IL-6 or IL-21, the Treg developmental pathway is abrogated, and instead T cells develop into Th17 cells. Only the combination of TGF-*β* plus IL-6/IL-21, but neither of them alone, induces a robust production of IL-17 by naive T cells [8, 9].

Therefore, IL-6 plays a pivotal role in dictating the balance between the generation of Tregs and Th17 cells. The mechanism by which IL-6 and IL-21 act as switch factors relies on the control of the Foxp3/ROR*γ*t balance [10, 11].

The reciprocal relationship between Tregs and Th17 cells is further supported by the results obtained in IL-6 knockout mice, which show a severe defect in the generation of Th17 cells and increased numbers of Tregs in the peripheral repertoire. Thus, IL-6 may be the most crucial factor in mediating the conversion of Foxp3^+^ T cells into Th17 cells *in vitro* and *in vivo.* The reexpression of the Th17 program in Foxp3^+^ cells appears to be a two-step process that includes downregulation of Foxp3 and release of ROR*γ*t from Foxp3-mediated inhibition [11, 12].

## 5. Treg, Th17, and Multiple Myeloma

 The role of Tregs in the biology of neoplastic diseases has been the subject of a large number of recent studies. However, many *in vitro* or *in vivo* results remain contradictory. For example, one study quantified numbers of Tregs in the peripheral blood of normal individuals versus patients with MGUS and MM and showed a significant reduction in the number of Treg cells, measured by Foxp3 expression in the patient group. These cells were described as dysfunctional and unable to suppress the proliferation of T lymphocytes in an organized manner [4]. On the other hand, another study compared the number and function of Tregs in the peripheral blood and bone marrow of normal individuals and patients with MM. They did not find a difference in the percentage of Treg cells between two compartments neither between the two groups of individuals [1].

Many studies about Th17 cells in humans have focused on patients with autoimmune diseases while there are very few studies on cancer patients. In the case of MM, recent publications have demonstrated increased number of Th17 cells in bone marrow in comparison with peripheral blood with different functional properties in these two compartments. This increase in Th17 cells was not observed in the bone marrow of patients with MGUS; however, numbers of Th17 cells were the highest in MM patients with lytic bone disease [13]. 

Our group has recently characterized the expression of Treg- and Th17-related genes in total bone marrow aspirates of MM and solitary plasmacytomas (SPs) to evaluate their potential as therapeutic targets in this disease. Total bone marrow seems to be a good source of samples for our study because (1) it reflects the “real” tumor bone marrow T-cell compartment, without manipulation of CD4 subpopulations by FACS or magnetic sorting and (2) normal and malignant plasma cells express no or very low levels of *Foxp3 or ROR-*γ*t* (data not shown), suggesting that expression of genes is representative for the respective CD4^+^ T cell subpopulation [14, 15].

**Figure 1:**
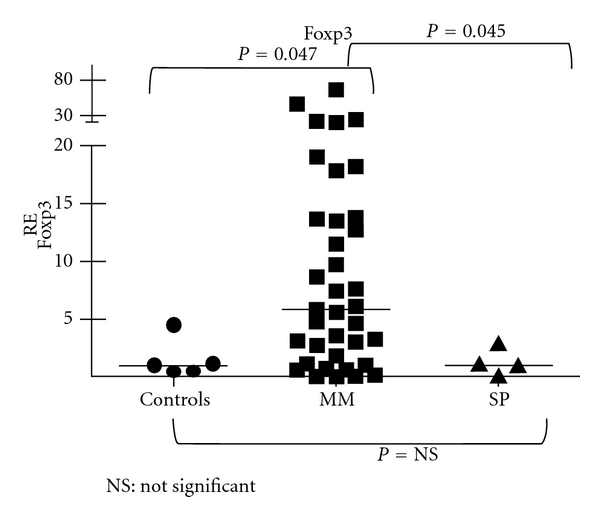
Relative expression (RE) of *Foxp3 *in bone marrow aspirates of myeloma, solitary plasmacytomas, and normal controls.

**Figure 2:**
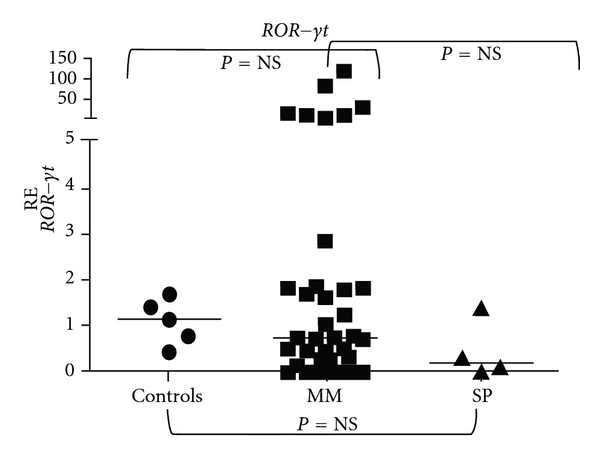
Relative expression (RE) of *ROR-*γ*t *in bone marrow aspirates of myeloma, solitary plasmacytomas, and normal controls.

When expressions of *Foxp3* and *ROR-*γ*t *genes were determined by quantitative real-time PCR (RQ-PCR) in bone marrow aspirates of 37 newly diagnosed MM patients, 4 newly diagnosed SPs, and 5 healthy controls (allogeneic transplant donors), *Foxp3* was overexpressed in 72% of MM cases. A 5.89-fold increase in *Foxp3* expression was observed in MM patients compared to controls (*P* = 0.047, Mann-Whitney test) (Figure 1). On the other hand, MM patients and controls showed equal levels of *ROR-*γ*t* expression and the difference between groups was not significant (Figure 2). Also, SP bone marrow aspirates showed *Foxp3* and *ROR-*γ*t *levels similar to controls. Overexpression of *Foxp3* in MM cases suggests an accumulation of immunosuppressive Tregs in the tumor environment and/or an immediate involvement of this gene in the development and progression of myeloma. Our results reinforce our hypothesis that therapeutic approaches that specifically target *Foxp3*-expressing Tregs may provide more focused treatment strategies for MM [14, 15]. Some studies have suggested that depletion of Treg cells with a possible “reprogramming” of these cells to proinflammatory Th17 cells could be a strategy of immunotherapy against tumors [16]. 

The immunomodulatory agents, such as lenalidomide, currently used as standard fist therapy, have a multifunctional action profile, with antiangiogenic activity, direct effects on myeloma cells, and alteration of the cytokine milieu within the BM microenvironment. These drugs exhibit potent costimulatory activity on primary T cells *in vitro* and lead to an increased IL-2 and IFN-y production following CD3 ligation. This effect may contribute to the reduction of the ratio CD4/8 and to an increase of natural killer cytotoxic cells. Lenalidomide is more potent than thalidomide in costimulating CD4 and CD8 T cells. Bortezomib is a proteasome inhibitor with significant clinical activity in MM, with also immunomodulatory with effects on the survival and function of lymphocytes and dendritic cells [3].

Further studies on the balance between Tregs and Th17 cells in malignancies such as MM are needed. Immunotherapy using single strategies in MM have shown little clinical efficacy, and there is a belief that a combined approach is required, as recently demonstrated in melanoma. In addition, results may further be improved by combining types of new immunotherapy with standard immunomodulatory agents (i.e., thalidomide, lenalidomide bortezomib) already being used for the treatment of myeloma.
